# B7H4 expression in tumor cells impairs CD8 T cell responses and tumor immunity

**DOI:** 10.1007/s00262-019-02451-4

**Published:** 2019-12-17

**Authors:** Linlin Zhou, Mei Ruan, Ying Liu, Yanyang Zhu, Deqiang Fu, Kunlin Wu, Qiuyu Zhang

**Affiliations:** 1 The School of Basic Medical Sciences, Fujian Medical University, Fuzhou, China; 2 Institute of Immunotherapy, Fujian Medical University, No. 1 Xuefu North Road, Shangjie Town, Minhou County, Fuzhou, 350122 China; 3 The First Affiliated Hospital of Fujian Medical University, No. 20 Chating Middle Road, Fuzhou, 350005 China

**Keywords:** B7 homolog 4, Tumor microenvironment, T cell, Immune suppression, Adoptive transfer

## Abstract

B7 homolog 4 (B7H4) is considered a negative regulator of immune responses, but the immunoregulatory role of B7H4 in the tumor microenvironment is not clear. Here, we assessed B7H4 expression cell types in human breast cancer tissues and addressed its potential mechanisms in the CD8 T cell immune response. The results from flow cytometry and immunohistochemistry demonstrated that B7H4 was highly expressed in 26 out of 30 (86.7%) breast invasive ductal carcinomas, and B7H4 surface expression on tumor cells was inversely correlated with CD8 T lymphocytes infiltration (*p* < 0.0001). In vivo, B7H4-overexpressing tumor cells showed enhanced tumor growth in immunocompetent mice with impaired CD8 T cell infiltration of the tumor. Further investigation showed that activation and expansion of CD8 T cells within the lymph nodes were suppressed in B7H4-overexpessing tumor-bearing mice. An in vitro killing assay showed that the cytotoxicity of CD8 T cells was inhibited in B7H4-overexpressing tumor cells. These findings suggest that B7H4 in tumor cells is a negative regulator of CD8 T cell activation, expansion and cytotoxicity, indicating that tumor cell-associated B7H4 might be a target for T cell-based cancer immunotherapy.

**Electronic supplementary material:**

The online version of this article (10.1007/s00262-019-02451-4) contains supplementary material, which is available to authorized users.

## Introduction

B7H4 (also referred to as B7x or B7S1) was found in 2003 and identified as a member of the B7 family of immune cosignaling molecules [2–4]. Aberrant B7H4 expression has been observed in various types of human cancer tissues and is thought to be correlated with advanced stages, poor prognosis, and overall patient survival [5–7]. However, the actual immunoregulatory role of B7H4 in the tumor microenvironment remains unclear. Most studies have shown that B7H4 is a negative regulator in T cell responses during antitumor immunity [4, 8–10], whereas one study demonstrated that B7H4 plays a promoting role in antitumor immunity [11]. These paradoxical results might be due to the difference in B7H4 expressing cell types and mouse tumor models used in the experiments. The B7H4 protein is known to be preferentially expressed in tumor cells of human cancer tissues [12–14]. However, it has also been detected in endothelial cells of small blood vessels and tumor-infiltrating myeloid cells [5, 15, 16]. Given what is now known about B7-H1 (PD-L1, programmed cell death 1 ligand 1) [17], examining B7H4 expression patterns and distribution in tumor microenvironment may provide additional insights into how B7H4 works in human cancer and immune response.

Hence, we analyzed B7H4 expression in tumor tissues, para-tumor and adjacent normal tissues and PBMC (peripheral blood mononuclear cells) derived from 30 patients with IDC (invasive ductal carcinomas), to assess the B7H4 expression pattern in the tumor site and its correlation with CD8 T cell infiltration. We further investigated the potential mechanisms of B7H4-overexpressing tumor cells in the CD8 T cell immune response, by the use of B7H4 overexpression mouse tumor models, T cell adoptive transfer and CD8 T cell killing assay.

## Materials and methods

### Patients and sample preparation

Tumor tissues, para-tumor and adjacent normal tissues and corresponding peripheral blood were obtained from 30 breast IDC patients who underwent surgery at the First Affiliated Hospital of Fujian Medical University from 2017 to 2018. None of these patients received preoperative radiotherapy or chemotherapy before surgery. Matching para-tumor tissues were defined as those ≤ 2.0 cm from the tumor edge and the adjacent normal tissues were procured at the most distant site from the resected specimen (≥ 5.0 cm from the tumor edge). Fresh tissue samples were cut and nonenzymatically dissociated, and single cells were prepared according to a previously described protocol [18]. PBMC were isolated using Ficoll-Paque PLUS (GE Healthcare, USA) according to the manufacturer’s instructions.

### Immunohistochemistry (IHC)

Human breast tissues were fixed and paraffin-embedded following standard procedures. The sections were immunolabeled with mouse anti-human CD8 (4B11, 1:100, AbD Serotec, UK) and mouse anti-B7H4 (6H3, 1:1000) antibodies, followed by anti-mouse peroxidase kit (ImmPRESS™, USA) labeling, respectively, according to the manufacturer’s instructions. Anti-B7H4 antibody (6H3) was produced in our lab.

### Tumor cell lines and tumor models

Human breast cancer cell lines (MCF-7, SKBR-3 and MDA-MB-468), mouse breast cancer cell lines (4T1, E0771), mouse glioma cell (GL261) and OVA transfection the EL4 thymoma cell (EG7) were cultured in RPMI-1640 or DMEM containing 10% FBS (Sigma-Aldrich). B7H4-overexpressing tumor cells (GL261-B7H4 and EG7-B7H4) were prepared by transfection of the pcDNA-mB7H4 plasmid encoding full-length mouse B7H4. C57BL/6 mice were anesthetized and 5 × 10^5^ GL261 or GL261-B7H4 tumor cells were injected into the right cerebral hemisphere of the brain at 4 mm depth below the surface of the scull using a Hamilton PB-600-1 Repeatable Dispenser. Tumor growth was monitored by bioluminescent imaging every 3–7 days using a Lumina XR imaging system (PerkinElmer), and the survival of mice was monitored daily. A total of 5 × 10^5^ EG7 or EG7-B7H4 cells were s.c. injected in the right flank of C57BL/6 mice or NSG mice. Tumor sizes were measured with digital calipers every 3 days and calculated using the equation (*l* + *w*)/2, where *l* and *w* refer to the larger and smaller dimensions, respectively. The mice were sacrificed as death for humane treatment after tumors reached a size of 2.0 cm in each dimension.

### OT-I adoptive transfer experiment

T cells were isolated from the spleens and lymph nodes of OT-I TCR (T cell receptor) transgenic mice by a Pan-Naive T Cell isolation kit (Stemcell Technologies, USA). Irradiated non-T cells (40 Gy) were cultured with 3 ng/ml chicken egg ovalbumin (OVA) peptide 257–264 (Peptides International) for 1 h, and then cocultured with OT-I T cells for 48 h. Mice were randomized into different treatment groups when EG7/EG7-B7H4 tumor diameters reached 5-8 mm and received an intravenous transfer of 2 × 10^6^ activated OT-I cells on day 10. IL-2 (2 × 10^4^ IU/mice) was i.p. administered to mice on days 10, 12 and 14. For the in vivo T cell expansion study, activated OT-I cells were labeled with 5 μM carboxyfluorescein diacetate succinimidyl ester (CFSE, Thermo Scientific, USA) before transfer, and then blood, spleen and lymph nodes were analyzed for flow cytometry.

### In vitro killing assay

To analyze OT-I cell cytotoxicity, EG7 or EG7-B7H4 cells (2 × 10^4^) were labeled with 3 μM CFSE as target cells, and then incubated with activated OT-I cells for 24 h at various effector-to-target ratios. To obtain tumor-specific cytotoxic T lymphocytes (CTLs), dendritic cells and CD8 T cells were isolated from the spleens and draining lymph nodes of GL261-bearing mice on day 7, respectively, using negative isolation microbeads (Miltenyi Biotec). CD8 T cells cocultured with tumor lysate pulsed-dendritic cells for 3 days. Viable CD8 T cells were purified with Lymphocyte-M (Cedarlane) and incubated with CFSE-labeled target cells (GL261/GL261-B7H4) for 24 h. Killing effect was evaluated by a cell death marker (LIVE/DEAD^®^ Fixable Dead Cell Stain kits, Thermo Scientific, USA) using flow cytometry. To observe the killing effect of CTLs under microscope, target cells (GL261/GL261-B7H4) were stained with 5 μM acetoxymethyl esters (AM, Thermo Scientific, USA) and coculture with tumor-specific T cells for 24 h. Live cell imaging and data analysis were performed using a Zeiss LSM 880 laser-scanning confocal microscope.

### Flow cytometry

TILs (tumor-infiltrating lymphocytes) were isolated from freshly resected tumor tissue using Gentle MACS mechanical dissociator containing lysis buffer (Miltenyi Biotec) and enriched according to the Lymphocyte-M manufacturer’s recommendations. ACK lysis buffer was used to lyse red blood cells. Cell suspensions from tissues were blocked with anti-mouse CD16/32 (TruStain fcX™, USA) before staining. Cells were stained with antibodies against mouse CD3, CD4, CD8, MHCII, CD137, CD40L, CD45.2, B7H4, TCR-Vβ5.1, CD25, Foxp3, IFN-γ, death marker and matched isotype controls, depending on the experiment. For intracellular cytokine staining, TILs were restimulated with 1 ng/ml OVA peptide 257–264 for 8 h in the presence of GolgiPlug (BD Bioscience, USA) before intracellular staining. Single cells from human tumor tissues were blocked with human FcR blocking reagent (Miltenyi Biotec, USA) and then stained with antibodies against human CD3, CD8, CD45 and B7H4, and with the death marker. These antibodies were obtained from eBioscience, Molecular Probes, or BD Biosciences. Samples were run on a BD FACSVerse™ (BD Biosciences, USA) and analyzed using FlowJo software (TreeStar, USA).

### Statistical analyses

Statistical analysis was conducted using GraphPad PRISM software (GraphPad Software, Inc. Version 6.03). Numerical data were expressed as the mean ± SEM except where otherwise noted. Statistical difference between groups was compared using Student’s *t* test or one-way ANOVA with Tukey’s or Dunnett’s multiple comparison test (tumor growth, phenotype comparisons). The log-rank and Wilcoxon tests were used to analyze the difference in survival time between groups. Values of *p* < 0.05 were considered indicative of significance.

## Results

### The association between B7H4 expression and CD8 T cell infiltration in the tumor tissues

**Fig. 1:**
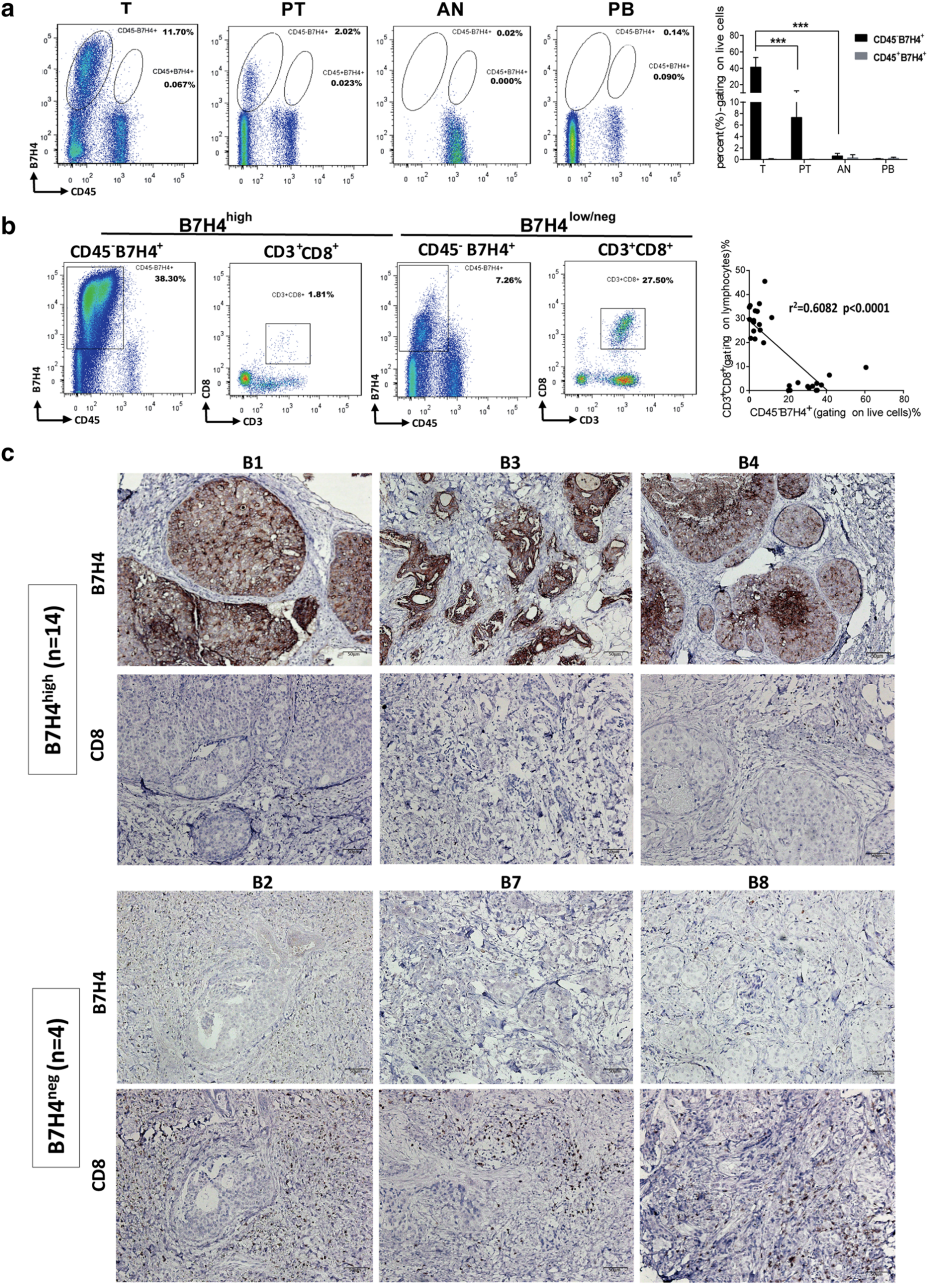
B7H4 expression and CD8 T cell infiltration in breast tumor tissues. **a** B7H4 expression was measured by flow cytometry in tumor tissues (T), para-tumor tissues (PT), adjacent normal tissues (AN), and peripheral blood mononuclear cells (PB) derived from patients with primary and metastatic ductal breast cancers. The percentage of CD45^−^B7H4^+^ cells and CD45^+^B7H4^+^ cells in different tissues (gating on live cells) were shown in representative FCM plots (left graph) and pooled from 8 cases with higher B7H4 expression in tumor tissues (CD45^−^B7H4^+^% > 30%, right graph). The results were expressed as the mean of the percentage of B7H4^+^ cells ± SEM in tissues or blood (***p < 0.001). **b** Percentage of CD45^−^B7H4^+^ cells (gating on live cells) and CD3^+^CD8^+^ (gating on lymphocytes) in breast tumor tissues were analyzed by flow cytometry. Representative FCM analysis plots from B7H4^high^ tumor tissues and B7H4^low/neg^ tumor tissues were shown in left graph. The correlation between the frequencies of CD45^−^B7H4^+^ cells and CD3^+^CD8^+^ cells was shown in the right graph (30 cases of invasive ductal carcinoma). **c** Immunohistochemical staining of B7H4 and CD8 in breast cancer tissues. Strong B7H4 protein staining was detected in the tubular epithelium of tumor sections from 14 cases of invasive ductal carcinoma with a low density of infiltrating CD8 T cells (upper graph). Negative expression of B7H4 with a high density of infiltrating CD8 T cells was detected in tumor sections from 4 cases (lower graph). B7H4^high^:high expression of B7H4; B7H4^low/neg^:low or no expression of B7H4

The clinical pathological features of 30 primary and metastatic ductal breast cancers (primary, 26.7%, 8 of 30 and metastases, 73.3%, 22 of 30) were listed in Supplementary Table 1. 26/30 cases of IDC (86.7%) were positive for B7H4 membrane-bound expression by flow cytometry. All B7H4 positive cells were only detected in the CD45-negative population from tumor and para-tumor tissues. The percentage of CD45^−^B7H4^+^ cells (gating on live cells) was higher in tumor tissues than that in para-tumor tissues (*p *< 0.001) (Fig. 1a). In addition, there was an inverse association between the proportion of CD45^−^B7H4^+^ cells and CD3^+^CD8^+^ T cells in tumor tissues of 26 IDC cases (*p *< 0.0001), especially in the cases expressing high levels of B7H4 (> 20% CD45^−^B7H4^+^ cells in live cell population, 14 cases, *p *= 0.0006) (Fig. 1b). Immunohistochemical staining revealed a high level of B7H4 expression on the cell surface and in the cytoplasm of tumor cells. The number of the CD8^+^ TILs was significantly lower in carcinoma cases with high levels of B7H4 expression in tumor cells (B7H4^high^) than in those with no B7H4 in tumor cells (B7H4^neg^) (Fig. 1c).

### B7H4 expression in tumor cell lines

**Fig. 2:**
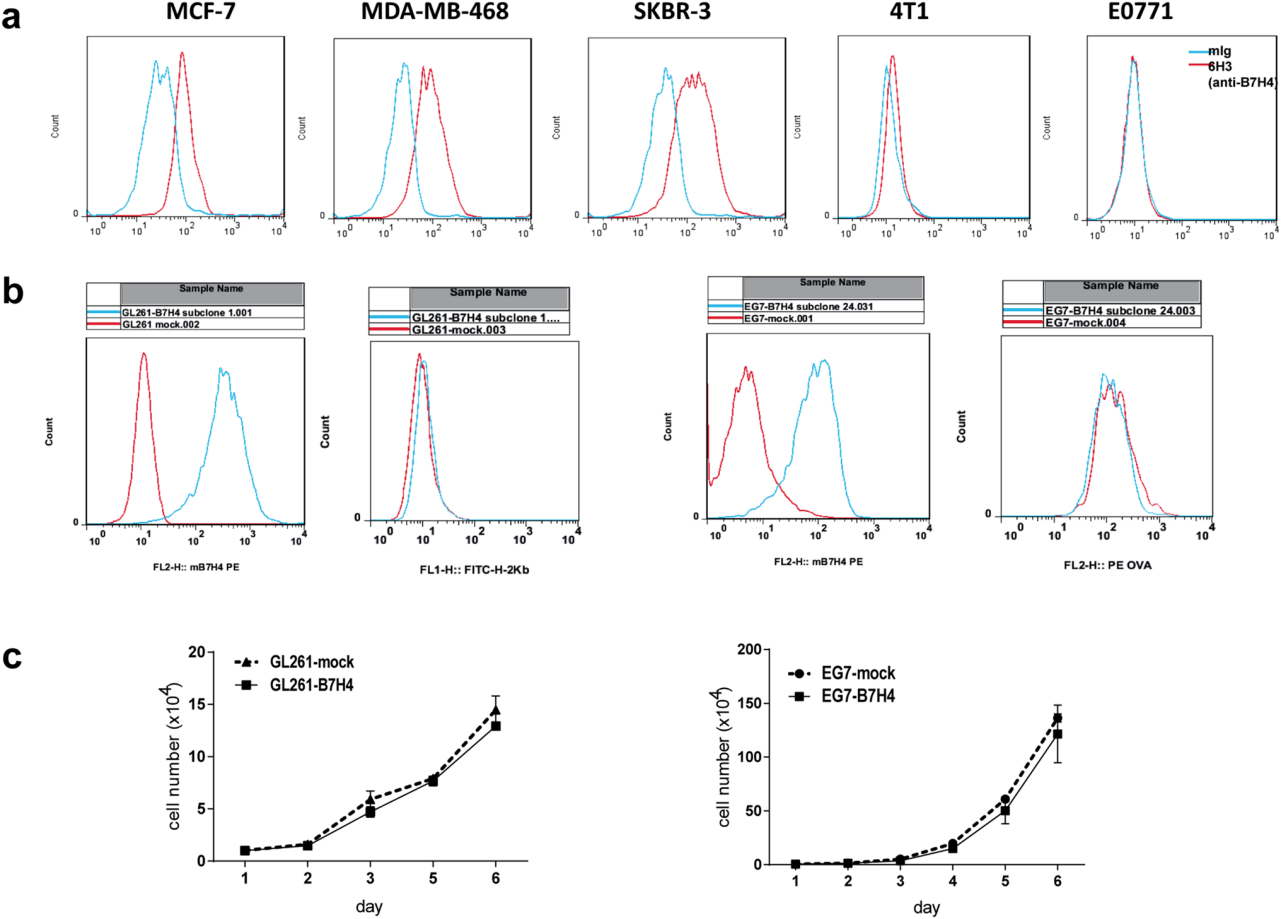
Preparation of B7H4-overexpressing mouse tumor cell lines. **a** The expression of B7H4 in human and mouse breast tumor cell lines was analyzed using anti-B7H4 antibodies by flow cytometry. **b** Two B7H4-overexpressing mouse tumor cell lines (GL261-B7H4 and EG7-B7H4) were confirmed by flow cytometry. MHC-I expression of GL261-B7H4 and OVA expression of EG7-B7H4 were analyzed using anti-MHC class I (H-2 Kb) and anti-OVA257-264 (SIINFEKL) peptide bound to a H-2 Kb monoclonal antibody, respectively. **c** Growth curve of two B7H4-overexpressing cell lines (GL261-B7H4 and EG7-B7H4) compared with control cells (GL261-mock cells or EG7-mock cells)

Surface B7H4 expression was detected on human breast cancer cell lines (MCF-7, SKBR-3 and MDA-MB-468), but not on mouse breast cancer cell lines (4T1 and E0771) (Fig. 2a). Stable B7H4-overexpressing GL261 and EG7 tumor cell line clones were selected. FCM (flow cytometry) analyses showed that B7H4 overexpression did not affect the expression level of MHC class I (H-2Kb) on GL261 cells (Fig. 2b, left graphs) and that OVA peptide bound to MHC class I on EG7 cells (Fig. 2b, right graphs). The cell growth curve showed no significant difference in cell proliferation between each of the two selected tumor cell lines and their respective controls in vitro (Fig. 2c).

### B7H4 overexpression potentiated tumor growth in immunocompetent mice

**Fig. 3:**
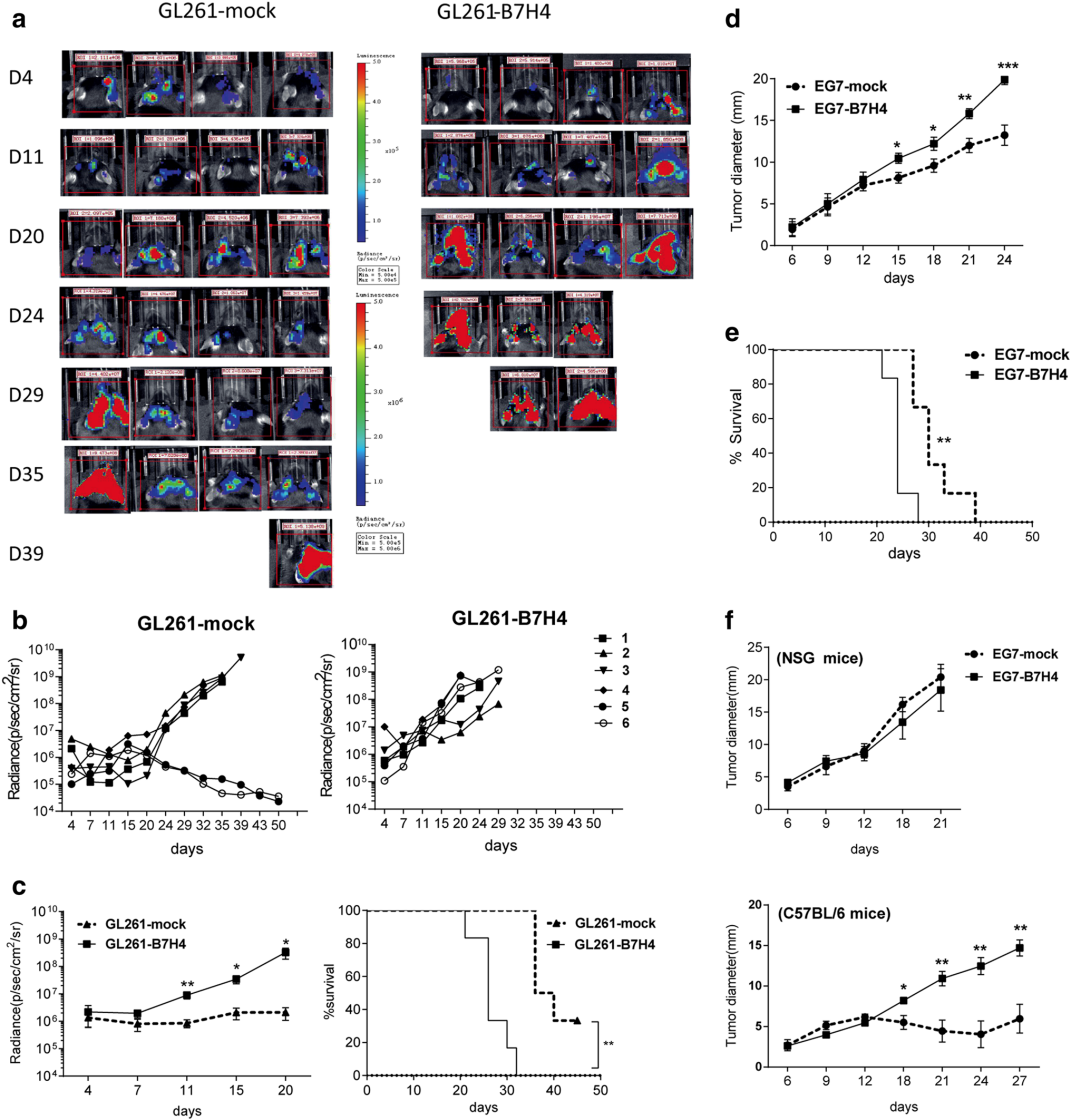
Differences in tumor growth rates between B7H4-overexpressing and control tumors. **a**, **b** In vivo bioluminescent imaging and computer-obtained luminescence (photons/second) of each tumor model mouse implanted with firefly luciferase-transgenic GL261-B7H4 cells or GL261-mock control cells. **c** Tumor growth and survival of GL261-B7H4 and GL261-mock bearing mice were recorded every 3–7 days; *n* = 6, one representative example of two independent experiments was shown. **d**, **e** Tumor volumes and survival curve for mice implanted with B7H4-overexpressing cells (EG7-B7H4) or EG7-mock cells, *n* = 5. **f** Tumor growth in C57BL/6 mice or NSG mice injected with B7H4-overexpressing cells (EG7-B7H4) and EG7-mock cells. These two types of tumor cells were collected on the same passage before injection, *n* = 5. Data are mean ± SD from a single experiment, representative of two or three independent experiments. *p < 0.05; **p < 0.01 and ***p < 0.001

B7H4-overexpressing transduced and control murine tumor cells were implanted into syngeneic C57BL/6 mice. Bioluminescence imaging showed that the growth of GL261-B7H4 tumor was faster than control tumor (GL261-mock) 11 days after tumor implantation (Fig. 3a, b, c left graph, *p *= 0.0095 on day 11). Correspondingly, GL261-B7H4-bearing mice died earlier than GL261-mock-bearing mice (Fig. 3c, survival curve, *p *= 0.0013). Similarly, B7H4 overexpression in EG7 tumor cells promoted tumor growth in immunocompetent mice resulting in decreased survival (Fig. 3d, e). However, when EG7-B7H4 and EG7-mock cells were implanted into immunodeficient NSG mice, there was no difference in tumor growth between the two types of tumor cells (Fig. 3f).

### B7H4 overexpression in tumor cells impaired CD8 T cell infiltration in a murine model

**Fig. 4:**
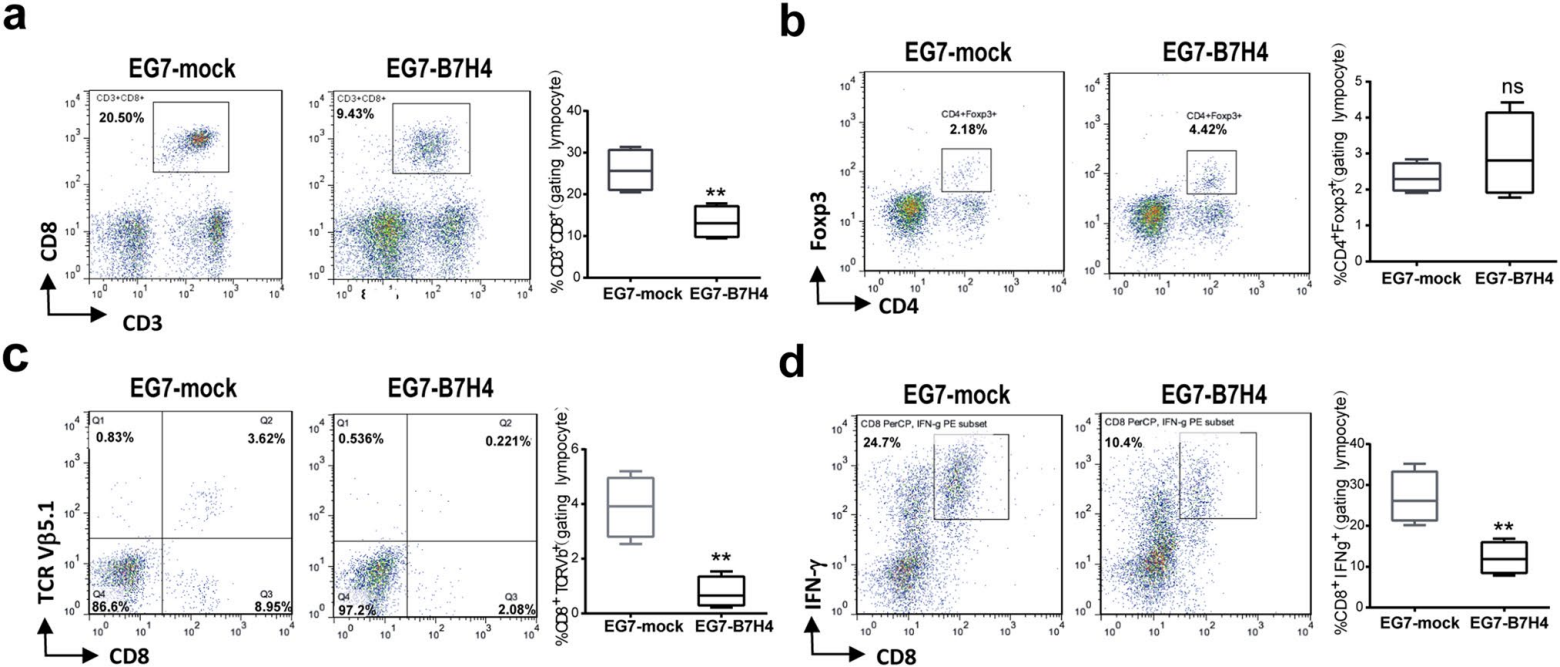
The analysis of tumor-infiltrating T cells in EG7-B7H4 and EG7-mock tumor tissues. EG7-B7H4 and EG7-mock cells were subcutaneously injected into C57BL/6 mice and tumor-infiltrating immune cells were analyzed by flow cytometry on day 21. The percentage of CD3^+^CD8^+^ T cells (**a**) and CD4^+^Foxp3^+^ T cells (**b**) in the lymphocyte population were shown as representative FCM plots in two groups (left graph), and pooled from two independent experiments (right graph, *n* = 6). **c** The percentages of TCRVβ5.1 staining in CD8 T cells derived from two groups were shown. **d** TILs were analyzed for cytokine production after stimulation with OVA-peptide, and the percentage of IFN-γ^+^CD8^+^ T cells in the lymphocyte population were shown. **p < 0.01

To further examine the impact of B7H4 on the immune response and tumor microenvironment, tumor infiltrating lymphocytes from EG7-mock and EG7-B7H4-bearing mice were analyzed on day 21. The frequency of CD3^+^CD8^+^ T cells and OVA-specific CD8 T cells (CD8^+^TCRVβ5.1^+^) in the total tumor-infiltrating immune cell population (CD45^+^ live cells) were significantly reduced in EG7-B7H4 tumors compared to EG7-mock tumors (Fig. 4a, c). To assess the IFN-γ production activity of CD8 T cells in tumor site, the intratumoral T cells from both tumors were stimulated with OVA-peptide for 8 h and intracellular staining of IFN-γ was performed. The data revealed that the frequency of CD8^+^IFN-γ^+^ T cells in EG7-B7H4 tumors was also lower than that in EG7-mock tumors (Fig. 4d). However, there was no significant difference in the frequency of CD4^+^Foxp3^+^Treg cells from both mice (Fig. 4b).

### Activation and expansion of CD8 T cells were suppressed in EG7-B7H4 tumor-bearing mice

**Fig. 5:**
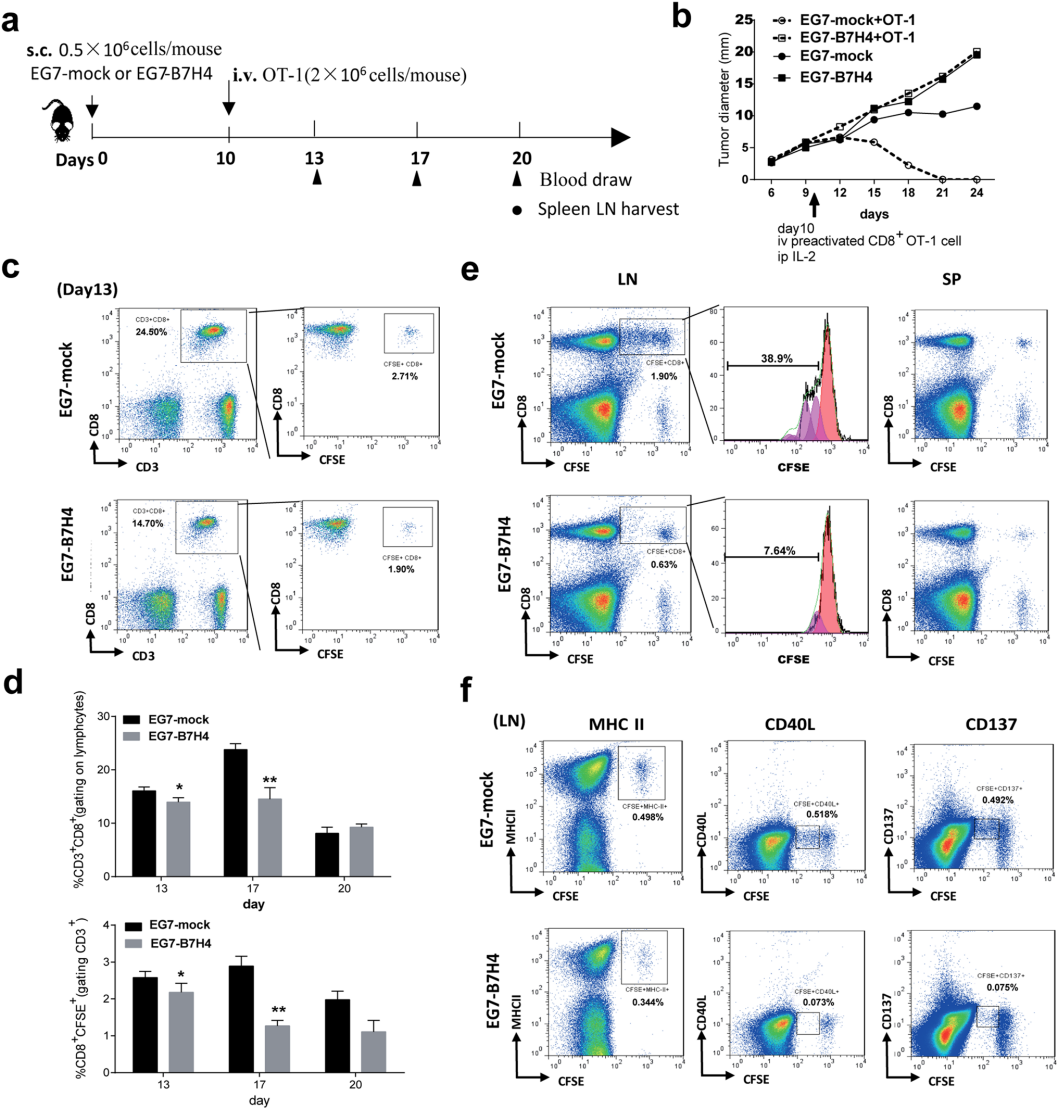
The antitumor role of adoptively transferred CD8 T cells in EG7-B7H4 and EG7-mock tumor-bearing mice. **a** Treatment schema. C57BL/6 mice were subcutaneously implanted with EG7-B7H4 or control tumor cells (EG7-mock) in the right flank. Tumor-established mice were randomized into two groups when tumors reached 5–8 mm in the largest diameter (typically on day 10 after inoculation). The two groups intravenously received adoptive transfer of activated OT-I cells and i.p. IL-2 on day 10, 12, 14. For flow cytometric analysis, OT-I cells were labeled with CFSE before transfer. **b** Tumor growth curve of four groups. **c** The frequency of CD3^+^CD8^+^ and CFSE^+^CD8^+^ T cells (gating on the lymphocyte population and CD3^+^CD8^+^ T cell population) in the peripheral blood of the treated mice (mice received adoptive transfer) on day 13 was shown in a representative FCM plot. **d** Average percentages of CD3^+^CD8^+^ and CFSE^+^CD8^+^ T cells on day 13, day 17 and day 20 pooled from two independent experiments (*n* = 6 mice per group). **e** The frequency of CFSE^+^CD8^+^ T cells (gating on the lymphocyte population) in the draining lymph node (LN) and spleen (SP) of the treated mice on day 20 were shown in representative FCM plots (left and right graph). The donor OT-I cell proliferation (gating on the CD8^+^ CFSE^+^ population) was analyzed by CFSE (middle graph). **f** The frequency of activated OT-I T cells (CFSE^+^MHCII^+^, CFSE^+^CD40L^+^, CFSE^+^CD137^+^) in the draining inguinal lymph node of the treated mice were analyzed on day 20. *p < 0.05; **p < 0.01

To assess whether B7H4-mediated immune suppression can be reversed by the adoptive transfer of antigen-specific CD8 T cells, EG7-B7H4 and EG7-mock tumor-bearing mice were intravenously injected with activated CD8 OT-I cells on day 10. Unexpectedly, exogenous antigen-specific T cells significantly inhibited EG7-mock tumor cell growth but there was no change in EG7-B7H4 tumor growth (Fig. 5b). To explain why the adoptive transfer of CD8 T cells had no effect on EG7-B7H4 tumor-bearing mice, OT-I T cells were labeled with CFSE before transfer, and then lymphocytes derived from peripheral blood, spleen and draining lymph node were analyzed on the indicated day according to the schedule (Fig. 5a). Three days after adoptive transfer (day 13), the frequency of CD8^+^CD3^+^ and CFSE^+^CD8^+^ OT-I cells in the peripheral blood were higher in EG7-mock tumor-bearing mice than in EG7-B7H4 tumor-bearing mice (Fig. 5c). In EG7-mock tumor-bearing mice, the frequency of peripheral CD8^+^CD3^+^ and CFSE^+^CD8^+^ OT-I cells were increased on day 17 and then decreased on day 20. However, in EG7-B7H4 tumor-bearing mice, the frequency of CFSE^+^CD8^+^ OT-I cells gradually decreased after transfer (Fig. 5d). In the draining lymph node, at least five cell divisions of CFSE^+^CD8^+^ OT-I cells were detected in EG7-mock tumor-bearing mice, while fewer divisions of OT-I cells were detected in EG7-B7H4 tumor-bearing mice (Fig. 5e left graphs). However, no cell division was detected in the spleen (Fig. 5e right graphs), and no cell division was found in CFSE labeled CD8^−^ T cells derived from peripheral blood, draining lymph node or spleen. These results suggested that the proliferation of CD8 T cells within the lymph node was suppressed in EG7-B7H4 tumor-bearing mice. To analyze the activation of proliferating T cells in the lymph node, we stained lymphocytes with antibodies against MHCII, CD40L and CD137. As expected, more activated lymphocytes (CFSE^+^MHCII^+^, CFSE^+^CD40L^+^ and CFSE^+^CD137^+^) were detected in EG7-mock tumor-bearing mice than in EG7-B7H4 tumor-bearing mice (Fig. 5f).

### B7H4-overexpressing tumor cells impaired the cytotoxicity of antigen-specific CD8 T cells

**Fig. 6:**
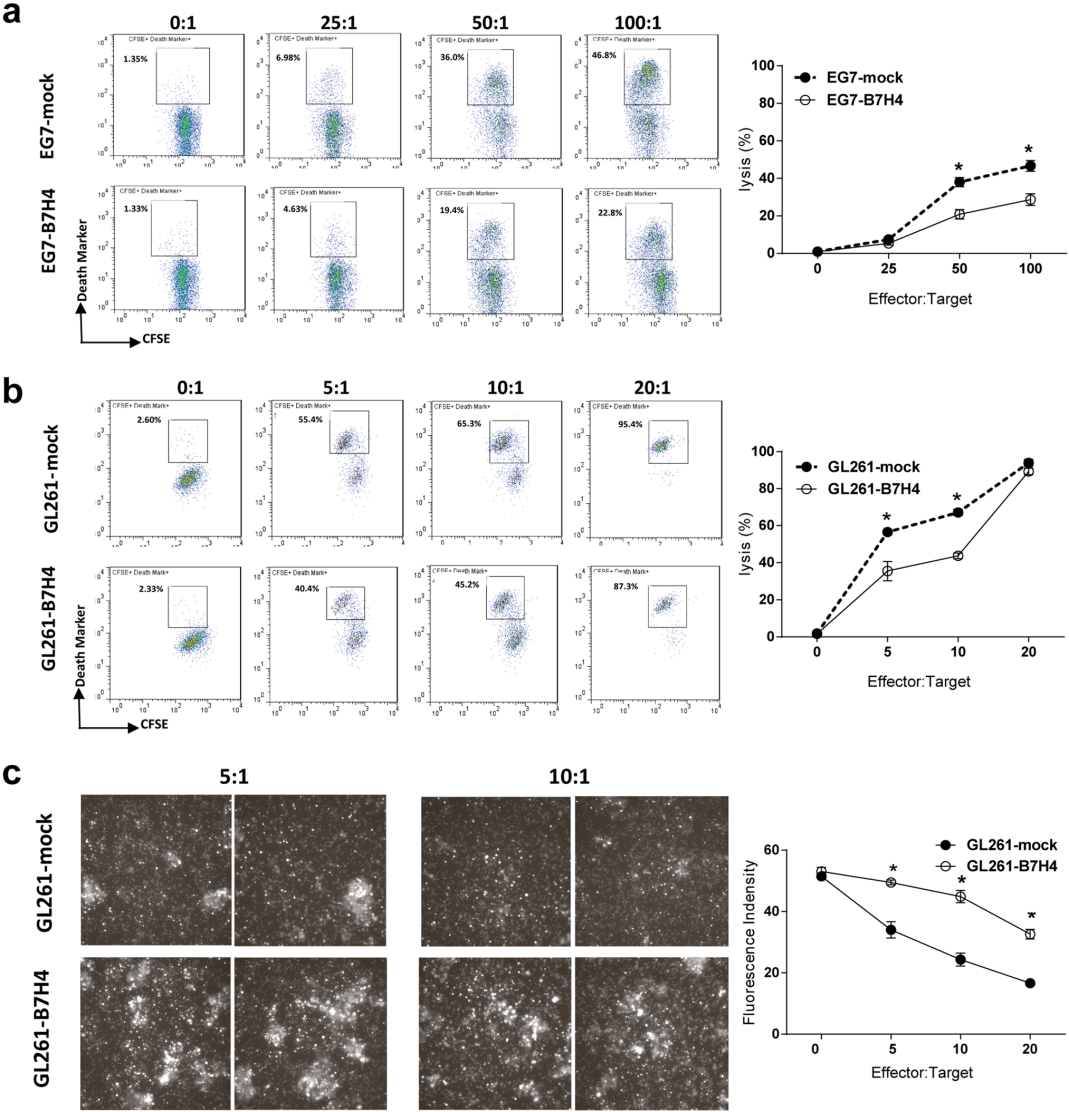
The cytotoxic effect of CD8 T cells on B7H4-overexpressing and control tumor cells. **a** The cytolytic activity of purified CD8 OT-I cells against CFSE labeled target cells (EG7-B7H4 or EG7-mock cells) was examined by FCM at the indicated effector:target cell ratios. The frequency of CFSE^+^Death-Marker^+^ tumor cells (gating on the CFSE-positive population) was shown in representative FCM plots (left graph), and the percentage of lysed cells was pooled from two independent experiments (right graph). **b** Purified CD8 T cells from GL261-bearing mice were harvested on day 7 and stimulated with γ-irradiated dendritic cells pulsed with GL261 cell lysate for 3 days. Viable T cells were purified by LymphoLyte-M and used at the indicated effector:target (E:T) ratios against CFSE labeled target cells (GL261-B7H4 or GL261-mock cells) for the killing assay as described above. **c** To observe the lysis of target cells under a microscope, target cells (GL261-B7H4 or GL261-mock cells) were stained with acetoxymethyl esters (live marker) and then cocultured with GL261 tumor-specific T cells for 24 h. Live cell imaging (left graph) and data analysis (right graph) were performed using a confocal microscope, (× 100 objective). The lysis percentage was calculated as follows:  % lysis = 100 × Death-Marker^+^CFSE^+^ target cells/total CFSE^+^ target cells. All assays were performed in triplicate. The data are representative of at least two independent experiments. *p < 0.05

The lack of an effect of the adoptive transfer of antigen-specific T cells on B7H4 tumor growth suggested that the cytotoxicity of CD8 T cells might be impaired in B7H4 tumor sites. An in vitro killing assay showed that compared to EG7-B7H4 cells, more EG7-mock cells in CFSE labeled population underwent lysis at higher effector: target ratios (50:1 and 100:1) (Fig. 6a). We next generated tumor specific cytotoxic CD8 T cells from GL261-bearing mice. Similarly, tumor specific CD8 T cells displayed a reduced killing ability toward GL261-B7H4 cells than to GL261-mock cells at optimal effector:target ratios (5:1 and 10:1) (Fig. 6b). Then in vitro imaging was used to confirm this phenotype. Correspondingly, more live cells were detected in GL261-B7H4 group than GL261-mock after coculture with CD8 T cells (Fig. 6c).

## Discussion

The T cell inhibitory mechanisms of B7H4 in the tumor microenvironment has not yet been resolved in detail. Kryczek suggested that human ovarian cancer-derived B7H4^+^ tumor-associated macrophage (TAM) suppress T cell proliferation and cytotoxicity [19]. Recently, Li et al. reported that B7H4 (B7S1) is highly expressed on tumor-infiltrating myeloid cells and promotes the exhaustion of activated CD8 TILs in mouse Hepa1-6 bearing mice [16]. It is noteworthy that B7H4 has been reported to be located in tumor cells but not immune cells in the majority of human cancer tissues by immunohistochemistry [6, 12, 14]. In the setting of antitumor immunity, cell surface B7H4 overexpression is thought to play a dominant role. In previous studies, B7H4 expression was determined by immunohistochemistry. Here, we analyzed B7H4 expression in a single cell suspension derived from fresh breast tumor, para-tumor and adjacent normal tissue samples by surface staining using flow cytometry. B7H4-positive cells were found in the CD45-negative cell population in breast tumor tissues, and no surface B7H4 expression was detected in tumor-associated macrophages (CD45^+^CD68^+^ cell population) and tumor vasculature (CD45^−^CD31^+^ cell population) (data not shown). IHC analysis further confirmed that B7H4 expression was largely restricted to the ductal epithelium of tumor tissues. Inconsistent with previous reports showing low intensity B7H4 staining in normal breast tissues [13, 20],we did not find B7H4 expression in adjacent normal breast tissues. Choi et al. also did not find B7H4 expression in a range of normal tissues including breast tissue [2]. These results indicate that B7H4 overexpression in breast cancer cells might be largely driven by oncogenic processes rather than antitumor immunity. More importantly, FCM results also showed an inverse correlation between B7H4 surface expression on breast tumor cells and the percentage of CD3^+^CD8^+^ cells, which was confirmed by IHC analysis. This inverse correlation has previously been reported in uterine endometrioid adenocarcinomas and non-small-cell lung cancer [21, 22]. These observations support the hypothesis that tumor-associated B7H4 may function as a negative regulator of the CD8 T cell immune response. The central distribution of B7H4 in breast tissues (high expression level in the center of the tumor tissues and low expression level in the para-tumor tissues) suggests that the inhibitory role of B7H4 on the CD8 T cell immune response is different from that of B7-H1 (PDL1). B7-H1 is known to be predominately expressed in the periphery of tumor cell conglomerates (interface distribution) and forms a shield at the periphery of the tumor tissue [17].

In an attempt to address the underlying immune mechanism of tumor surface B7H4 expression on CD8 T cell function, we sought to develop tumor models in immunocompetent mice using mouse tumor cell lines with surface B7H4 expression. Nevertheless, no constitutive surface B7H4 expression was found in all available mouse tumor cell lines; therefore, two B7H4-overexpressing clones were generated in GL261 (mouse glioma) and EG7 (an OVA-transfected EL4 thymoma) cell lines. Intriguingly, B7H4 overexpression induced tumor growth in immunocompetent mice but not in immunodeficient NSG mice, indicating that overexpression of B7H4 in tumor cells might be a mechanism by which tumors could avoid eliciting an antitumor immune response. In our present study, no B7H4 positive cell was detectable in the CD45-positive cell population of human breast cancer tissues and mouse tumor tissues derived from GL261- and EG7-bearing mice. Hence, we hypotheized that B7H4 overexpression in tumor cells might play a dominant role in the inhibition of T cell antitumor responses.

Although in vitro studies using immobilized B7H4 Ig fusion protein or cell-associated B7H4 have suggested that B7H4 might deliver an inhibitory signal to T cells, thereby abrogating CD8 T cell proliferation and cytotoxicity [4], to data, little is known about how B7H4 impairs the T cell immune response in vivo. Hence, we analyzed the intratumoral T cells on day 21 in EG7-B7H4- or EG7-bearing mice. Our data revealed that B7H4 overexpression in tumor cells decreased the frequency of antigen-specific CD8 T cells in the tumor site and reduced IFN-γ production. To further investigate the dysfunction of effector CD8 T cells in EG7-B7H4-bearing mice, activated T cells (OT-I T cells) were adoptively transferred into EG7-B7H4- and EG7-mock tumor-established mice. As expected, adoptive transfer of OT-I cells inhibited EG7-mock tumor growth but had no effect on EG7-B7H4 tumor growth. Intriguingly, we found that OT-I cells transferred into the mice implanted with EG7-B7H4 did not divide, while CFSE^+^CD8^+^ OT-I cells transferred into mice implanted with EG7-mock cells went through more than five divisions within 10 days. T cell surface marker analysis showed that all the divided CD8 OT-I cell were activated T cells (MHCII^+^CD137^+^CD40L^+^) in EG7-mock tumor-bearing mice. Collectively, these results suggest that the ability of antigen-specific CD8 activation and expansion were impaired in mice implanted with B7H4-overexpressing tumors. This finding offers an explanation for the failure of adoptive transfer of the OT-I cells to inhibit EG7-B7H4 tumor growth. Furthermore, B7H4 cell surface expression on both EG7 and GL261 mouse tumor cell lines suppressed CD8 T cell cytotoxicity in vitro.

In conclusion, the unique expression of B7H4 in tumor cells and its inverse relationship with the number of tumor-infiltrating CD8 T lymphocytes in breast invasive ductal cancer tissues supports the inhibitory role of tumor surface B7H4 on T cell immune responses. In a mouse tumor model, B7H4 overexpression on the tumor surface fosters tumor growth in immunocompetent mice by suppressing the activation, expansion and cytotoxicity of CD8 tumor-specific T cells. These findings provide new insight into the role of tumor-associated B7H4 in impairing T cell-mediated immunity and shaping the tumor microenvironment.

## Electronic supplementary material

Below is the link to the electronic supplementary material.

Supplementary material 1 (PDF 286 kb)

## Abbreviations

B7H4: B7 homolog 4
CFSE: Carboxyfluorescein diacetate succinimidyl ester
CTL: Cytotoxic T lymphocyte
FCM: Flow cytometry
IHC: Immunohistochemistry
IDC: Invasive ductal carcinomas
PBMC: Peripheral blood mononuclear cell
TCR: T cell receptor
TAM: Tumor associated macrophage
TIL: Tumor infiltrating lymphocyte
