# Supplementary material for: B7H4 expression in tumor cells impairs CD8 T cell responses and tumor immunity
Source: Cancer Immunol Immunother. 2019 Dec 17;69(2):163–74. doi: 10.1007/s00262-019-02451-4 (PMC7000514; doi:10.1007/s00262-019-02451-4)
Supplement: Supplementary file 1 — Supplementary material 1 (PDF 286 kb) [file 262_2019_2451_MOESM1_ESM.pdf]

Supplementary Table 1. Patients' characteristics

Clinico-pathological Factors (N=30)		N	(%)	Clinico-pathological Factors (N=30)		N	(%)
Age				ER status			
	≤50	12	40.0		Negative	8	26.7
	>50	18	60.0		Positive	22	73.3
Stage					Unknown	0	0.0
	I	5	16.7	PR status			
	II	19	63.3		Negative	10	33.3
	III	6	20.0		Positive	19	63.3
Grade					Unknown	1	3.3
	1	2	6.7	Her-2 status			
	2	21	70.0		Negative	10	33.3
	3	7	23.3		Positive	18	60.0
No. lymph node					Unknown	2	6.7
	0	8	26.7	Ki-67 status			
	1-3	18	60.0		≤20%	14	46.7
	>3	4	13.3		>20%	16	53.3
tumor size (cm)				Molecular classification			
	≤2	8	26.7		Luminal A	13	43.3
	2-5	21	70.0		Luminal B	12	40.0
	>5	1	3.3		$ERBB2^+$	5	16.7

ER: Estrogen receptor; PR: Progesterone receptor.
